# The challenges and opportunities in the expanding horizons of microbiota transplant therapies

**DOI:** 10.1080/19490976.2025.2559032

**Published:** 2025-11-03

**Authors:** Alexander Khoruts

**Affiliations:** Department of Medicine, Division of Gastroenterology, Hepatology, and Nutrition, University of Minnesota, Minneapolis, MN, USA; Center for Immunology, University of Minnesota, Minneapolis, MN, USA; BioTechnology Institute, University of Minnesota, St. Paul, MN, USA

## Microbiota-based therapeutics require well thought-out and nuanced regulatory policies

Over the last decade, fecal microbiota transplantation (FMT), long relegated to the periphery of medical practice, has emerged as a mainstream therapeutic option for the increasingly common and difficult challenge of recurrent *Clostridioides difficile* infections.^1,2^ The US Food and Drug Administration (FDA) has taken an early notice of this development. In May 2013, at a public workshop, the FDA announced that it classified FMT as a drug and a biologic (an unapproved drug at the time). This regulatory decision has set off a race among several drug companies to introduce a first-in-class FMT-based product to capture early advantages in claiming market share and patent exclusivity. Until then, the FDA had introduced an ‘enforcement discretion’ policy, which allowed FMT to be used for treatment of *C. difficile* infections not responding to antibiotics alone. During this period, the regulation of FMT in Europe has been fragmented and highly variable among individual countries, ranging from no regulation to that of a human cell or tissue product (HCT/P) to a medicinal product. However, after a prolonged deliberative process that included extensive input from multiple stakeholders, the European Union has implemented the Substances of Human Origin (SoHO) Regulation, which includes fecal microbiota along with breast milk and other substances or cells derived from the human body, living or not.

In this issue of *Gut Microbes*, Hoffmann and colleagues review the contrasting histories of FMT regulation in the US and Europe.^3^ The implications of regulatory decisions have had a major impact on patient care and the development of FMT-based therapeutics. In the US, approval of FMT-based ‘drugs’ led to cessation of distribution of FMT material by OpenBiome, a nonprofit that for a decade was the major source of centrally produced cryopreserved fecal microbiota under a policy of ‘enforcement discretion.’ Unfortunately, the high cost of the FDA-approved commercial products and patchy insurance coverage have limited their uptake in clinical practice. In addition, most pediatric patients and those with severe and fulminant *C. difficile* infection lost access to FMT because FDA restricted commercial product approval to *prevention* of *C. difficile* infection recurrence, rather than *treatment*, and use in adults only. Poor product performance in the marketplace and ongoing litigation between different drug companies over intellectual property associated with FMT-based products have also dampened investor interest in the development of microbiota-based therapeutics. Furthermore, the hard-wired manufacturing processes inherent to the drug approval process have limited further innovation.^4^

In contrast to the FDA, the European SoHo Regulation has tried to ensure the greatest patient access to FMT, while preserving the potential for FMT-based medicinal product development. In addition, while the FDA has emphasized the standardization of the processing steps in manufacturing, the European SoHo Regulation highlighted donor safeguards and volunteer participation. Some of the most common questions asked by patients do pertain to donor selection processes. Notably, although approval by regulatory agencies is reassuring to most patients, clinical providers are also increasingly faced with skepticism and questioning of authority.^5^ In the current issue, Kabage and colleagues describe the unique logistical and ethical challenges associated with the management of stool donor programs.^6^ The demands placed on stool donors, including rigorous testing regimes and scheduled supervised donations, are intense. However, using monetary inducements as the major tool for donor recruitment and retention creates a moral hazard that can compromise the integrity of the stool donor program. Independent, nonprofit organizations that engage with stool donors as partners in pursuing humanitarian goals may be better suited for building stool donor programs. Hopefully, as the field continues to grow and mature, best practices will also develop alongside, just as they have in the sphere of blood banking.

## Complexity of FMT is a ‘feature not a bug’ of the live biotherapeutics products class of drugs

The FDA designation of FMT as a drug has generated a certain sense of befuddlement among investigators and clinical providers. After all, the human gut microbiota is integral to its host physiology and adapted to its host through co-evolution. Of course, all live biotherapeutic products, including FMT, are fundamentally different from traditional drugs, such as small molecules and protein biologics. Understanding their pharmacology requires the incorporation of previously distant scientific disciplines, such as microbial ecology, and a revision of the standard germ theory of disease to allow entire microbial communities, rather than individual microbes, to play major roles in disease pathogenesis.

The compositional complexity of FMT clearly sets it apart from traditional drugs. While this complexity has been unsettling for some investigators and regulators, it mirrors the natural state of fecal microbiota, enabling adaptation and resilience to environmental changes and insults. Currently, there is no evidence that simple defined microbial consortia can outperform FMT in any specific clinical scenario. Sufficient early engraftment of a few chosen bacterial strains may be able to block the recurrence of *C. difficile* infection by engaging critical mechanisms, such as restoration of secondary bile acid metabolism and short-chain fatty acid production.^7^ However, this approach relies more heavily upon (unregulated) environmental sources to replenish missing microbes in the antibiotic-depleted microbiome.^8,9^ Unfortunately, healthcare-associated environments harbor reservoirs of antibiotic-resistant pathobionts that may exploit such vulnerabilities.^8,10^ Long-term studies are needed to compare the microbiome content of antibiotic resistance genes and virulence factors in patients treated with simple therapeutic microbial consortia versus complete donor-derived communities.

An opposing strategy to defined consortia aimed at achieving compositional uniformity has been mixing donations from multiple donors in preparation of FMT material.^11,12^ This approach allows for greater consistency in relative abundances among major bacterial taxa and ensures the presence of the desired microbial groups. Interestingly, although the microbial richness of FMT preparations made from pooled donations is very high, it quickly becomes comparable to the level measured in individual donors.^12^ This observation suggests that there are severe ecological and physiologic constraints on the microbial diversity in the human gut, which may be driven by inter-strain competition, composition of nutrient flow, immunologic, and other still unsuspected factors. Whether the resulting microbial community, which is composed of the fittest survivors, is functionally superior in terms of its resilience and benefits to the host, remains an open question.

## Development of FMT-based drugs requires a pharmacologic framework

**Figure 1.**
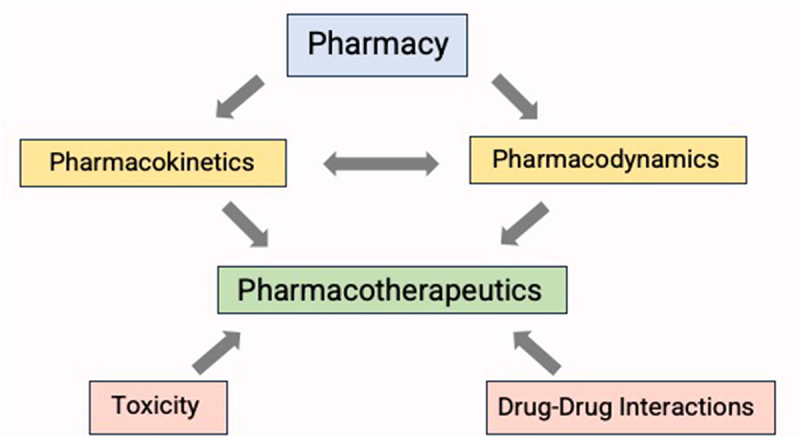
Elements of pharmacology. safe and effective use of any drug requires knowledge of its drug composition and formulation, including the excipients (pharmacy), understanding of its pharmacokinetics and pharmacodynamics, potential toxicities and drug-drug interactions. Pharmacotherapeutics is the integration of all these elements, which enables the clinical provider to administer the drug safely, effectively, and rationally to achieve the desired therapeutic outcome.

Despite the pronounced differences in the intrinsic complexity of FMT-based products relative to traditional drugs, the pharmacological framework remains essential for the advancement of this new frontier in therapeutics development. This framework encompasses different aspects of the formulation and route of administration (pharmacy), the fate of the drug in the body (pharmacokinetics), the body response to the drug (pharmacodynamics), as well as considerations of drug-drug interactions and toxicities (Figure 1).

A common criticism of FMT is its lack of standardization, which includes both the natural variation in microbiome composition among healthy individuals as well as differences in preparation protocols. However, while FMT does not allow compositional uniformity of the formulation, preparation of FMT-derived products can be standardized and operationalized using Good Manufacturing Practices protocols. Microbiota can be purified from stool and quantified in terms of the number and viability of donor microbes. FMT can be formulated as cryopreserved liquid suspensions or freeze-dried preparations, using cryo- or lyo-protectants, respectively.^13–15^ Oral administration allows easier repeated administration, which is increasingly used in protocols targeting non-*C. difficile* indications. In addition, varying the characteristics of capsule disintegration can be used to target different parts of the gastrointestinal tract.

**Figure 2.**
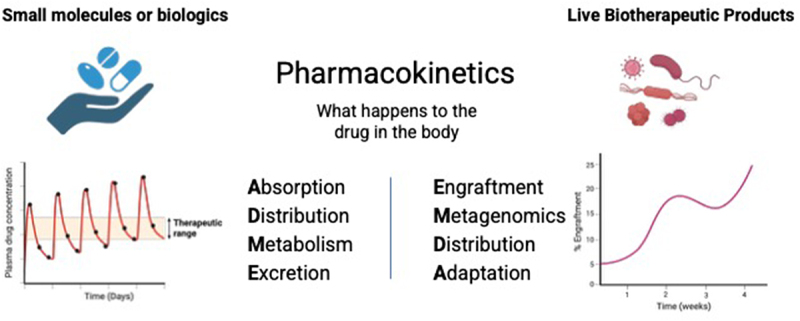
Pharmacokinetics of live biotherapeutic products. The classical adme parameters (absorption, distribution, metabolism, elimination) used to describe the pharmacokinetics of traditional drugs, including small molecules and protein biologics, are not well-suited for live biotherapeutic products. A modified set of criteria (emda) is proposed here, which stands for engraftment, microbiome, distribution, adaptation. engraftment measurements can be considered at the level of the donor microbial community or individual microbial strains of interest. ‘Metagenome’ refers to the collective genetic material associated with the donor microbiota. It is also important to understand the distribution of donor microbiota within the various complex microenvironments of the gastrointestinal tract, which cannot be fully captured by analyzing fecal samples alone. Finally, the donor microbes may undergo adaptation to the new environment. For example, bacterial donor strains may alter their pattern of gene expression or acquire new traits via horizontal gene transfer. The figure was created with BioRender.com.

At first glance, the basic elements of drug pharmacokinetics – Absorption, Distribution, Metabolism, Excretion – seem to be a poor fit for FMT or any live biotherapeutic products targeting the gut microbiome (Figure 2). Traditional drugs are metabolized and eliminated after entering the body. In contrast, FMT generally leads to (1) sustained colonization of the gut by donor microbial strains, (2) introduction of new functional traits due to acquisition of new genes, (3) differential distribution of donor microbes within the various microenvironment of the gastrointestinal tract, and (4) ultimate adaptation of donor microbiota to the new host environment driven by strain competition and exchange of mobile genetic elements. Thus, a comprehensive description of the pharmacokinetics of FMT and all live biotherapeutic products should ultimately include the following basic elements: Engraftment, Metagenome, Distribution, Adaptation (Figure 2), which mirror the classic ADME parameters.

**Figure 3.**
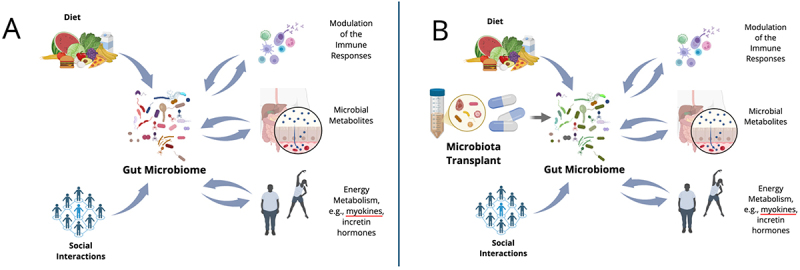
Pharmacodynamics of live biotherapeutic products. A. The gut microbiome interacts with various aspects of the host physiology via signaling delivered by microbial metabolites and direct stimulation of immune receptors. Conversely, the host responses impact the functions of the gut microbiota. Diet and exercise modulate the relative abundances of different microbial taxa and their activities. New microbial strains enter from the environment, e.g., diet and social interactions, and compete with the indigenous microbiota. However, while these environmental influences affect the activity of the microbiota, they have only minor effects on the microbiome composition. B. transplantation of the donor microbiota from a healthy donor restructures the gut microbiome. Some indigenous bacterial strains, including those with high burden of antibiotic resistance and virulence factor genes, are eliminated via competitive niche exclusion and other mechanisms. The restructured microbiome delivers new signaling along the many axes of microbiota host interactions. These effects occur in the context of continued environmental inputs. Adjunctive measures, such as diet, physical activity, and household environments may contribute to the success of microbiota transplant therapies in ensuring the stability and optimal functionality of the new microbiome. The figure was created with BioRender.com.

Pharmacodynamics refers to the mechanistic understanding of the effects of a drug on the body. Investigations of FMT pharmacodynamics may include functional effects on the microbiome itself, e.g., content of antibiotic resistance and virulence factor genes, as well as the effects on the host physiology (Figure 3). The gut microbiome is an organ-like structure in the body that produces metabolites that interact with the enteric and central nervous systems,^16^ affect production of incretin hormones, e.g., GLP-1,^17,18^ and calibrate systemic and mucosal immune responses.^19^ Detailed understanding of how FMT or other live biotherapeutic products can beneficially engage these mechanisms is critical to the development of next-generation therapeutics. Conversely, understanding of gut microbiota-host interactions can also minimize potential toxicities of FMT-based interventions, which may not be limited to the possibility of introducing potential pathogens.^20^

Finally, as with any therapeutics, clinicians need to consider potential drug-drug interactions. Some of these may seem obvious, e.g., co-administered antibiotics may negate FMT benefits. However, given the potential roles of the gut microbiome in the metabolism of many drugs,^21,22^ clinicians may need to consider the possible impact of FMT on other medications with a narrow therapeutic index, e.g., warfarin, organ transplant anti-rejection medications.^23,24^ The data on these potential interactions remains very limited and observational. However, it may be prudent to consider the possibility of such interactions and implement closer monitoring of certain medications.

## Evolving DNA computational and sequencing technologies are being employed to characterize FMT pharmacokinetics

FMT may be viewed as a collision of donor and recipient microbial communities, which takes place in a space that is also open to microbes in environment. The resulting complexity creates a formidable challenge for characterizing and quantifying the FMT pharmacokinetics. Several papers in this issue of *Gut Microbes* address this fundamental problem. Herman and colleagues describe the common basic framework of the current approaches and highlight three major features: *Community Coalescence*, *Indicator Feature* tracking, and long-term *Resilience*.^25^ Most of the pharmacokinetic investigations of FMT interventions continue to center their investigations on community-level changes in the microbiome and tracking donor microbiota over time. However, individual microbial strain dynamics within intestinal ecological micro-niches may deviate substantially from the community averages, and Jeremiah Faith discusses progress being made in tracking the dynamics of individual donor strains.^26^ Currently, there remains an urgent need for efficient and accurate methods in tracking donor microbiota engraftment over time. In this issue, Hoops and colleagues describe a new cost-efficient analytic pipeline, MAGEnTa, which uses metagenome-assembled genomes directly from donor and pre-treatment metagenomic data, without relying on an external database.^27^ This new tool should prove useful in tracking both community and strain engraftment dynamics.

The sequencing technologies and bioinformatic analytics for studying microbial communities continue to advance rapidly. Some of the next-generation methodologies, such as long-read metagenomics, high-throughput chromosome conformation capture (Hi-C or proximity ligation shotgun metagenomics), and droplet digital PCR, enable improved genome assembly, taxonomic resolution, linkage of genes to their microbial hosts, and accurate quantification of specific sequences. In this issue of *Gut Microbes*, Bryson and colleagues applied proximity ligation shotgun metagenomics to track the donor bacteria and associated plasmids and bacteriophages in a cohort of patients treated with oral capsule FMT for recurrent *C. difficile* infection.^28^ In contrast to traditional shotgun metagenomics, which relies on indirect sequence-based evidence to infer associations between extrachromosomal elements and specific bacteria, this technique directly links genes to their specific bacterial hosts by physically cross-linking DNA molecules close to each other within cells prior to digestion and sequencing of the chimeric DNA fragments. The enhanced level of resolution should be helpful in tracking the dynamics of functional trait acquisition by specific bacterial hosts encoded by phages and plasmids in the context of FMT. One example of a potential application could be the analysis of FMT-associated shifts in the resistome and mobilome linked to *clinically important* bacterial taxa, such as Extended-Spectrum Beta-Lactamase-producing Enterobacteriaceae and Vancomycin-Resistant *Enterococci*.

## Mechanistic understanding is necessary to develop FMT into next-generation therapeutics

The growing appreciation of the roles that the gut microbiome plays in host hormonal metabolism, calibration of immune responses, and modulation of neurologic development and function has generated considerable interest in FMT beyond treating difficult *C. difficile* infections. However, restructuring an intact but potentially dysfunctional (or ‘dysbiotic’) microbiome to a healthier composition is a more challenging objective than merely restoring a depleted microbiome in patients with recurrent *C. difficile* infections. Achieving this will require the development of next-generation live biotherapeutic products and the establishment of effective usage protocols. Various FMT-based clinical trials have the potential to provide deeper mechanistic insights, next-generation product development, even though it remains unclear whether they will retain FMT complexity or emerge as defined and limited consortia of specific bacterial strains.

Progress depends on constructing effective FMT protocols that yield signals of clinical benefit or at least promising changes in relevant biomarkers. Several manuscripts in this issue of *Gut Microbes* explore certain variables pertaining to FMT protocol design, including donor and recipient selection, FMT dosing, and adjunct therapies, such as diet.^29–32^ Notably, although greater engraftment of donor microbiota is often a reasonable initial objective, there is not always a good correlation between the extent of engraftment and clinical outcomes.^33^ Thus, deep mechanistic understanding of microbial community dynamics and microbiota-host interactions is going to be critical in the journey toward more effective live biotherapeutics. Two manuscripts in this issue undertake an in-depth mechanistic exploration of microbiota-based therapeutics development. Barrios Steed and colleagues consider opportunities in using non-antibiotic therapeutic strategies, including FMT, in reducing the growing threat posed by multidrug-resistant organisms.^34^ Nagayama and colleagues build their vision of precision microbiota therapies for inflammatory bowel diseases through the synthesis of descriptive microbiome studies and analysis of FMT trials.^35^

## Microbiota transplant therapies emerge as the next chapter in FMT development that targets indications beyond *C. difficile* infections

The demonstration of donor microbiota engraftment in a patient with recurrent *C. difficile* infections led to a rapid adoption of the term ‘Fecal Microbiota Transplant’ or ‘FMT.’^36,37^ The term has experienced multiple challenges. ‘Fecal Microbiota *Transfer*’ was preferred by some to emphasize the uncertainty over the extent of donor microbiota engraftment and the mechanisms of action.^38^ ‘Intestinal Microbiota Transplant’ (IMT) was proposed as an alternative to avoid the nonsensical versions such as ‘Fecal Transplant’ (‘Fecal’ merely describes the source of the donor microbiota), ‘Stool Transplant,’ and various scatological derivatives that sometimes enter clinical practice.^39^ Nevertheless, the term ‘FMT’ has remained resilient. In fact, although engraftment of donor microbiota is only partial even in patients with recurrent *C. difficile* infection, the repair of antibiotic-induced microbiome depletion is both rapid and dramatic, while the term ‘transplant’ acknowledges the critical contribution of human stool donors and encompasses many elements of transplantation medicine and.

Importantly, although a single administration of FMT is highly effective in breaking the recurrence cycle of *C. difficile* infection, much more complex protocols are increasingly being used in FMT-inspired strategies targeting the gut microbiome for most other indications. In fact, the decimated microbiota in patients with recurrent *C. difficile* infections is almost unique in the extent of repeated and intentional antibiotic treatments prior to the infusion of donor microbiota. Thus, a single FMT is typically sufficient to achieve substantial engraftment of donor microbiota and clinical cure. However, antibiotic conditioning and/or repeated administrations of donor microbiota markedly enhance restructuring of the microbiome in most non-*C. difficile* conditions.^33^ In addition, the extent of donor microbiota engraftment does not always correlate with clinical benefits,^33^ and additional mechanisms, e.g., stimulation of immune receptors during the passage of donor microbiota, may play important roles in mediating clinical effects.^40^

Kang and colleagues originally proposed a new term, ‘Microbiota Transfer Therapy’ or ‘MTT,’ in their initial autism trial.^41^ The group has since modified it to ‘Microbiota Transplant Therapy.’^42^ The common and distinguishing features of FMT and MTT are summarized in Table 1. The term ‘transplant’ in ‘MTT’ acknowledges the elements of transplant medicine in using human donor microbiota, while the term ‘therapy’ captures the complexity of the regimen. The protocol described by Kang et al. includes initial conditioning with vancomycin, which builds on an early observation of transient improvement in behavioral symptoms in patients with regressive-onset autism,^43^ which is then followed by a purgative step to wash out the antibiotic, a loading dose of donor microbiota, and a prolonged administration of a low-dose maintenance donor microbiota regimen. Future development of MTT for autism may include optimizing dosing regimens optimization and adjunctive nutritional measures to favor microbiota most beneficial to the host.

**Table 1.** Fecal microbiota transplant (fmt) versus microbiota transplant therapy (MTT).

	FMT	MTT
Source of microbiota	Human donor stool	Human donor stool
Regimen	One or two administrations of donor microbiota	Complex regimen that may include one or more additional elements, such as intentional antibiotic conditioning, bowel purgative, repeated administrations of donor microbiota, and adjunctive prebiotic supplements or dietary therapies
Indication	Primarily *C. difficile* Infections	Most non-*C. difficile* indications^1^
Recipient microbiota	Severely depleted by antibiotics	Variable antibiotic exposure during the preceding clinical course
Target	Colon microbiota	Various compartments within the gastrointestinal tract and different host organ systems
Donor selection	Any healthy donor without recent antibiotic exposure	Donor selection besides qualifying health criteria is likely to play a significant role in achieving clinical benefit

^**1**^Treatment of fulminant *C. difficile* infections typically requires multiple sequential administrations of donor fecal microbiota.

While the mechanistic objective of MTT in autism may be restructuring the gut microbiome to alter the production of neuroactive microbial metabolites in the gut, the mechanistic objective in other indications may be entirely different. For example, in hepatic encephalopathy it may be greater lactulose utilization and elimination of ammonia. In patients undergoing checkpoint immunotherapy, it is enhanced anti-tumor activity without triggering checkpoint inhibitor colitis. In infectious disease, it may be decrease in the burden of multidrug-resistant organisms in the gut. Each indication is likely to have a different MTT regimen that is personalized to the clinical objective and individual patient factors..

## References

[1] Kelly CP, LaMont JT. Clostridium difficile — more difficult than ever. N Engl J Med. 2008;359(18):1932–8. doi: 10.1056/NEJMra0707500. 18971494

[2] Borody TJ, Khoruts A. Fecal microbiota transplantation and emerging applications. Nat Rev Gastroenterol Hepatol. 2012;9(2):88–96. doi: 10.1038/nrgastro.2011.244. 22183182

[3] Hoffmann DE, Javitt GH, Kelly CR, Keller JJ, Baunwall SMD, Hvas CL. Fecal microbiota transplantation: a tale of two regulatory pathways. Gut Microbes. 2025;17(1):2493901. doi: 10.1080/19490976.2025.2493901. 40302307 PMC12054926

[4] Khoruts A, Hoffmann DE, Palumbo FB. The impact of regulatory policies on the future of fecal microbiota transplantation. J Law Med Ethics. 2019;47(4):482–504. doi: 10.1177/1073110519897726. 31957587

[5] Chou WYS, Gaysynsky A. A relationship-centered approach to addressing mistrust. J Commun Healthcare. 2023;16(4):320–323. doi: 10.1080/17538068.2023.2258683. 37732639

[6] Kabage AJ, Haselhorst PJ, Khoruts A. Donor-centric administration of the stool donor program is vital to its feasibility and patient safety. Gut Microbes. 2025;17(1):2508950. doi: 10.1080/19490976.2025.2508950. 40530459 PMC12184138

[7] Menon R, Bhattarai SK, Crossette E, Prince AL, Olle B, Silber JL, Bucci V, Faith J, Norman JM. Multi-omic profiling a defined bacterial consortium for treatment of recurrent Clostridioides difficile infection. Nat Med. 2025;31(1):223–234. doi: 10.1038/s41591-024-03337-4. 39747680

[8] Khoruts A. Can FMT cause or prevent CRC? Maybe, but there is more to consider. Gastroenterology. 2021;161(4):1103–1105. doi: 10.1053/j.gastro.2021.06.074. 34224741

[9] Sarkar A, McInroy CJA, Harty S, Raulo A, Ibata NGO, Valles-Colomer M, Johnson KVA, Brito IL, Henrich J, Archie EA, et al. Microbial transmission in the social microbiome and host health and disease. Cell. 2024;187(1):17–43. doi: 10.1016/j.cell.2023.12.014. 38181740 PMC10958648

[10] Chng KR, Li C, Bertrand D, Ng AHQ, Kwah JS, Low HM, Tong C, Natrajan M, Zhang MH, Xu L, et al. Cartography of opportunistic pathogens and antibiotic resistance genes in a tertiary hospital environment. Nat Med. 2020;26(6):941–951. doi: 10.1038/s41591-020-0894-4. 32514171 PMC7303012

[11] Malard F, Loschi M, Huynh A, Cluzeau T, Guenounou S, Legrand F, Magro L, Orvain C, Charbonnier A, Panz-Klapuch M, et al. Pooled allogeneic faecal microbiota MaaT013 for steroid-resistant gastrointestinal acute graft-versus-host disease: a single-arm, multicentre phase 2 trial. EClinicalMedicine. 2023;62:102111. doi: 10.1016/j.eclinm.2023.102111. 37654670 PMC10466244

[12] Paramsothy S, Kamm MA, Kaakoush NO, Walsh AJ, van den Bogaerde J, Samuel D, Leong RWL, Connor S, Ng W, Paramsothy R, et al. Multidonor intensive faecal microbiota transplantation for active ulcerative colitis: a randomised placebo-controlled trial. Lancet. 2017;389(10075):1218–1228. doi: 10.1016/S0140-6736(17)30182-4. 28214091

[13] Hamilton MJ, Weingarden AR, Sadowsky MJ, Khoruts A. Standardized frozen preparation for transplantation of fecal microbiota for recurrent Clostridium difficile infection. Am J Gastroenterol. 2012;107:761–767. doi: 10.1038/ajg.2011.482. 22290405

[14] Staley C, Hamilton MJ, Vaughn BP, Graiziger CT, Newman KM, Kabage AJ, Sadowsky MJ, Khoruts A. Successful resolution of recurrent Clostridium difficile infection using freeze-dried, encapsulated fecal microbiota; pragmatic cohort study. Am J Gastroenterol. 2017;112(6):940–947. doi: 10.1038/ajg.2017.6. 28195180 PMC5552199

[15] Sadowsky MJ, Matson M, Mathai PP, Pho M, Staley C, Evert C, Weldy M, Khoruts A. Successful treatment of recurrent Clostridioides difficile infection using a novel, drinkable, oral formulation of fecal microbiota. Digestive Dis Sci. 2024;69(5):1778–1784. doi: 10.1007/s10620-024-08351-7. 38457115

[16] Needham BD, Kaddurah-Daouk R, Mazmanian SK. Gut microbial molecules in behavioural and neurodegenerative conditions. Nat Rev Neurosci. 2020;21:717–731. doi: 10.1038/s41583-020-00381-0. 33067567

[17] Fasano A. The physiology of hunger. NEJM Evid. 2025;392(4):372–381. doi: 10.1056/NEJMra2402679. 39842012

[18] Zeng Y, Wu Y, Zhang Q, Xiao X. Crosstalk between glucagon-like peptide 1 and gut microbiota in metabolic diseases. mBio. 2024;15:e0203223. doi: 10.1128/mbio.02032-23. 38055342 PMC10790698

[19] Takeuchi T, Nakanishi Y, Ohno H. Microbial metabolites and gut immunology. Annu Rev Immunol. 2024;42(1):153–178. doi: 10.1146/annurev-immunol-090222-102035. 38941602

[20] DeFilipp Z, Bloom PP, Torres Soto M, Mansour MK, Sater MRA, Huntley MH, Turbett S, Chung RT, Chen Y-B, Hohmann EL. Drug-resistant E. coli bacteremia transmitted by fecal microbiota transplant. N Engl J Med. 2019;381(21):2043–2050. doi: 10.1056/NEJMoa1910437. 31665575

[21] Weersma RK, Zhernakova A, Fu J. Interaction between drugs and the gut microbiome. Gut. 2020;69:1510–1519. 32409589 10.1136/gutjnl-2019-320204 PMC7398478

[22] Zimmermann M, Zimmermann-Kogadeeva M, Wegmann R, Goodman AL. Mapping human microbiome drug metabolism by gut bacteria and their genes. Nature. 2019;570:462–467. doi: 10.1038/s41586-019-1291-3. 31158845 PMC6597290

[23] Xue L, Singla RK, Qin Q, Ding Y, Liu L, Ding X, Qu W, Huang C, Shen Z, Shen B, et al. Exploring the complex relationship between vitamin K, gut microbiota, and warfarin variability in cardiac surgery patients. Int J Surg. 2023;109(12):3861–3871. doi: 10.1097/JS9.0000000000000673. 37598356 PMC10720796

[24] Gabarre P, Loens C, Tamzali Y, Barrou B, Jaisser F, Tourret J. Immunosuppressive therapy after solid organ transplantation and the gut microbiota: bidirectional interactions with clinical consequences. Am J Transpl. 2022;22(4):1014–1030. doi: 10.1111/ajt.16836. 34510717

[25] Herman C, Barker BM, Bartelli TF, Chandra V, Krajmalnik-Brown R, Jewell M, Li L, Liao C, McAllister F, Nirmalkar K, et al. A review of engraftment assessments following fecal microbiota transplant. Gut Microbes. 2025;17(1):2525478. doi: 10.1080/19490976.2025.2525478. 40605266 PMC12233830

[26] Faith JJ. Assessing live microbial therapeutic transmission. Gut Microbes. 2025;17:2447836. doi: 10.1080/19490976.2024.2447836. 39746875 PMC12931690

[27] Hoops SL, Moutsoglou D, Vaughn BP, Khoruts A, Knights D. Metagenomic source tracking after microbiota transplant therapy. Gut Microbes. 2025;17:2487840. doi: 10.1080/19490976.2025.2487840. 40229213 PMC12005403

[28] Bryson S, Nelson B, Grove J, Reister E, Liachko I, Auch B, Graiziger C, Khoruts A. Use of proximity ligation shotgun metagenomics to investigate the dynamics of plasmids and bacteriophages in the gut microbiome following fecal microbiota transplantation. Gut Microbes. 2025;17(1). doi: 10.1080/19490976.2025.2559019. PMC12439552 40948444

[29] Ghani R, Chrysostomou D, Roberts LA, Pandiaraja M, Marchesi JR, Mullish BH. Faecal (or intestinal) microbiota transplant: a tool for repairing the gut microbiome. Gut Microbes. 2024;16:2423026. doi: 10.1080/19490976.2024.2423026. 39499189 PMC11540080

[30] Teigen LM, Hoeg A, Zehra H, Shah P, Johnson R, Hutchison K, Kocher M, Lin AW, Johnson AJ, Vaughn BP. Nutritional optimization of fecal microbiota transplantation in humans: a scoping review. Gut Microbes. 2025;17(1):2446378. doi: 10.1080/19490976.2024.2446378. 39772953 PMC11730610

[31] Moutsoglou D, Ramakrishnan P, Vaughn BP. Microbiota transplant therapy in inflammatory bowel disease: advances and mechanistic insights. Gut Microbes. 2025;17:2477255. doi: 10.1080/19490976.2025.2477255. 40062406 PMC11901402

[32] Harris SC, Bajaj JS. Interaction of the gut-liver-brain axis and the sterolbiome with sexual dysfunction in patients with cirrhosis. Gut Microbes. 2025;17:2446390. doi: 10.1080/19490976.2024.2446390. 39764615 PMC12931715

[33] Podlesny D, Durdevic M, Paramsothy S, Kaakoush NO, Högenauer C, Gorkiewicz G, Walter J, Fricke WF. Identification of clinical and ecological determinants of strain engraftment after fecal microbiota transplantation using metagenomics. Cell Rep Med. 2022;3(8):100711. doi: 10.1016/j.xcrm.2022.100711. 35931074 PMC9418803

[34] Barrios Steed D, Koundakjian D, Harris AD, Rosato AE, Konstantinidis KT, Woodworth MH. Leveraging strain competition to address antimicrobial resistance with microbiota therapies. Gut Microbes. 2025;17:2488046. doi: 10.1080/19490976.2025.2488046. 40195644 PMC11988218

[35] Nagayama M, Gogokhia L, Longman RS. Precision microbiota therapy for IBD: premise and promise. Gut Microbes. 2025;17:2489067. doi: 10.1080/19490976.2025.2489067. 40190259 PMC11980506

[36] Khoruts A, Dicksved J, Jansson JK, Sadowsky MJ. Changes in the composition of the human fecal microbiome after bacteriotherapy for recurrent Clostridium difficile-associated diarrhea. J Clin Gastro. 2010;44:354–360. doi: 10.1097/MCG.0b013e3181c87e02. 20048681

[37] Bakken JS, Borody T, Brandt LJ, Brill JV, Demarco DC, Franzos MA, Kelly C, Khoruts A, Louie T, Martinelli LP, et al. Treating Clostridium difficile infection with fecal microbiota transplantation. Clin Gastroenterol Hepatol. 2011;9(12):1044–1049. doi: 10.1016/j.cgh.2011.08.014. 21871249 PMC3223289

[38] Konig J, Siebenhaar A, Hogenauer C, Arkkila P, Nieuwdorp M, Noren T, Ponsioen CY, Rosien U, Rossen NG, Satokari R, et al. Consensus report: Faecal microbiota transfer - clinical applications and procedures. Aliment Pharmacol Ther. 2017;45(2):222–239. doi: 10.1111/apt.13868. 27891639 PMC6680358

[39] Khoruts A, Brandt LJ. Fecal microbiota transplant: a rose by any other name. Am JGastroenterol. 2019;114(7):1176–1176. doi: 10.14309/ajg.0000000000000286. 31205129

[40] Crothers JW, Chu ND, Nguyen LTT, Phillips M, Collins C, Fortner K, Del Rio-Guerra R, Lavoie B, Callas P, Velez M, et al. Daily, oral fmt for long-term maintenance therapy in ulcerative colitis: results of a single-center, prospective, randomized pilot study. BMCGastroenterol. 2021;21(1):281. doi: 10.1186/s12876-021-01856-9. PMC8268596 34238227

[41] Kang DW, Adams JB, Gregory AC, Borody T, Chittick L, Fasano A, Khoruts A, Geis E, Maldonado J, McDonough-Means S, et al. Microbiota transfer therapy alters gut ecosystem and improves gastrointestinal and autism symptoms: an open-label study. Microbiome. 2017;5(1):10. doi: 10.1186/s40168-016-0225-7. 28122648 PMC5264285

[42] Takyi E, Nirmalkar K, Adams J, Krajmalnik-Brown R. Interventions targeting the gut microbiota and their possible effect on gastrointestinal and neurobehavioral symptoms in autism spectrum disorder. Gut Microbes. 2025;17:2499580. doi: 10.1080/19490976.2025.2499580. 40376856 PMC12087657

[43] Sandler RH, Finegold SM, Bolte ER, Buchanan CP, Maxwell AP, Vaisanen ML, Nelson MN, Wexler HM. Short-term benefit from oral vancomycin treatment of regressive-onset autism. J ChildNeurol. 2000;15(7):429–435. doi: 10.1177/088307380001500701. 10921511

